# The Social Epidemic
*Prostitution considered in its Moral, Social, and Sanitary Aspects in London and other large Cities; with proposals for the mitigation and prevention of its attendant evils. By Wm. Acton, M.R.C.S. London: 1857.


**Published:** 1858-01

**Authors:** 


### THE SOCIAL EPIDEMIC.*

THE subject of the work in hand has been so freely discussed in the periodicals of the day, that it would be mere prudery to offer any apology for drawing attention to its contents. No one at all acquainted with the state of modern society will be at all disposed to deny that the evil of which the book treats is of immense magnitude, for its roots extend deep into the soil on which our present social fabric rests, whilst its branches overshadow the privacies of our domestic life. It is an evil which leaves its traces discoverable by the eye of the physician among all classes of the people, and from which neither riches form a safeguard nor poverty a protection.

It is an ungracious task to hold up the mirror and show the form and pressure of the time; for, while it shows us our railways, telegraphs, and leviathan steamers, our monster meetings for preachings, for social science, and for the fine arts, it shows us withal a shady side of vice and sensuality, which in magnitude can only be likened to that of the accursed cities of antiquity. We sing an everlasting "Io Pæan" on progress, on the march of intellect, and on national prosperity. We plume ourselves on subjugating the elements, we overcome the winds and the waves, and try to annihilate time and space. Now and then, with great timidity, some chivalrous man of science ventures gently to raise the veil

("For in the fatness of these pursy times,
Virtue itself of vice must pardon beg,")

which screens our social habits, and gives us a view of the obscene mysteries, which are being constantly enacted in our streets, and a picture of manners comparable only with the wildest saturnalia of the worst of pagan times. In the book before us, we have the death's head and cross bones brought into the feasts of good society; and enough is related to mar the complacency of the most well-bred conventionalist.

Whoever knows anything of London life, will at once confess that the evil which the author has endeavoured to describe he has not at all attempted to exaggerate. The actual misery and disease need no description. They describe themselves, and may be seen in the streets, unless kept out of sight by the police. Those who require facts and figures for conviction, must read the statistics of venereal diseases. The author marshals his facts which look like a funeral procession; he preaches a

---

* Prostitution considered in its Moral, Social, and Sanitary Aspects in London and other large Cities; with proposals for the mitigation and prevention of its attendant evils. By WM. ACTON, M.R.C.S. London: 1857.

funeral oration on the death of national chastity. He does more; he gives us a *post mortem* examination, and demonstrates that our seeming virtue is little other than a whited sepulchre,

> "Whiles rank corruption, mining all within,
> Infects unseen."

In treating of the causes of prostitution, the author frequently quotes Mr. Mayhew's letters to the *Morning Chronicle*, in which the demoralisation of children is described as taking place in the low lodging houses of London and elsewhere. It will suffice to say, that these letters describe scenes of juvenile precocity in vice, which the narrator confesses cannot be detailed in print. Mr. Acton observes:

"The extreme youth of the junior portion of the street walkers is a remarkable feature of London prostitution, and has been the subject of much comment by foreign travellers who have published their impressions of social London. Certain quarters of the town are positively infested by juvenile offenders, whose effrontery is more intolerably disgusting than that of their elder sisters. It is true these young things spring from the lowest dregs of the population; and from what I can learn of their habits, their seduction, if seduction it can be called, has been effected with their own consent by boys no older than themselves, and is an all but natural consequence of promiscuous herding,—that mainspring of corruption among our lower orders."

Under such circumstances, it can hardly excite surprise that a sort of practical communism should prevail among the lower orders. The value of chastity is not appreciated by them as it should be. Thus the author:

"It may, I dare say, be objected, that in preceding pages I have lent myself to a gross slander upon the public at large, by setting at so grievous a discount the popular estimation of virtue and propriety. But it is not to a fervid imagination, but hard memory and the experience of our profession, that I owe the preceding facts and analysis; and when I consider what I have written, I confess I can see no single statement or opinion that can surprise the major part of readers conversant with London, although their juxtaposition be new, and favour startling inferences. If we consult the experiences of the clergy, who are the best of authorities upon the social condition of both urban and manufacturing communities, or men who, like the Brothers Mayhew, have sifted to the dregs the lower orders in capital cities, and in the metropolis particularly, we find that female honour by no means holds its theoretical position in public esteem." (p. 71.)

So, from the evidence of people best capable of judging, it would appear that we are "drifting" into a sea of socialism. As to the extent to which prostitution prevails, it is impossible to form any approximative calculation; thus

"Mr. Colquhoun, at that time magistrate at the Thames Police Court, rated them at 50,000 some sixty years ago. The Bishop of Exeter spoke of them as reaching 80,000. Mr. Talbot, secretary of a society for the protection of young females, made the same estimate." (p. 15.)

It can easily be understood that this is a profession which, whether it be practised for love or money, is usually carried on as privately as possible, and that it is only the open and avowed courtesans who are constantly walking the streets, or who inhabit houses of ill fame, that are likely to come under the cognisance of the authorities. But this class forms but an insignificant portion of the female community who are living in more or less open concubinage. As the wages accorded to female labour in slopwork, millinery, dressmaking, bookbinding, artificial flower making, &c., are not such as to afford an honest subsistence, many girls who have not other means beside their work are partly compelled by want, partly encouraged by example, and partly tempted by pleasure, to leave the steep and thorny way to heaven, for the primrose path of dalliance.

It would appear almost a work of supererogation to quote figures to prove that prostitution prevails to an enormous extent; however, those exquisite optimists who desire to learn the truth would do well to look into Mr. Acton's book, where they will find the evidence of all classes. The clergy, the police, the reputable householders and municipal corporations, the medical men and hospitals, all testify to the extent of the moral disease. We shall therefore take it as an established fact, that all the accounts we hear are not exaggerated.

The only excuse for bringing this subject before the eye of the general public, is the hope that reformation may be brought about. The publication of the details of a revolting traffic; the natural history of the professed woman of the streets; the description of the means and appliances of professed bawds and their modes of communication with their patrons, is not an unmixed good to the community. Although a scientific investigator may be called upon to detail the results of his experience, and although the *litterateur* may find his end in giving photographic representations of filth and low debauchery, and although this may stimulate the really good to exert themselves for the amelioration of vice, yet, to the sensual, the vicious, the young and inexperienced, these scientific books thus popularised are too liable to be converted into mere guide-books to vice, or to afford amusement to the prurient fancy of the depraved; and thus, as it were, they hold a candle to the devil, by suggesting means and appliances for vicious indulgences which otherwise might never have been thought of. The following is quoted from Mr. Mayhew's letters to the *Morning Chronicle.*

"I used to work at slopwork, at the shirtwork, the fine full-fronted white shirts, I got 2¼d. each for 'em. There were six button holes, four rows of stitchings in the front, and the collar and wrist-bands stitched as well. By working from *five o'clock in the morning until midnight* each night, I might be able to do seven in the week, these would bring me in 17½d. for my *whole week's labour*. Out of this the cotton must be taken, and that came to 2d. every week, and so left me 15½d. to pay rent, and living, and buy candles with. I was single, and received some little help from my friends, still it was impossible for me to live, *I was forced to go out of a night to make out my living.*" (p. 25.)

Now we have no hesitation in saying, that the gentleman who noted this down was imposed upon by some canting girl of the town. Any woman who knows anything of needlework will say at once that this is a ridiculous fable. Any woman may earn at least eight or ten shillings in the workrooms of London, if she be not right down idle; besides, if the girl in question went out at night to eke out a living, we can hardly fancy her close working for nineteen hours a day, and for six days, for the small sum of one shilling and threepence half-penny. We were hardly prepared for such implicit faith, in a gentleman whose humanitarianism seems especially devoted to young thieves, prostitutes, and ticket-of-leave men.

We cannot follow Mr. Acton in his *Harlot's Progress,* nor accompany him in his peregrinations through their *Homes and Haunts of London;* through *dress houses*; houses in which prostitutes lodge; introducing houses; accommodation houses; casinos; pleasure gardens; and the streets. But we proceed to the consideration whether any means can be suggested for the mitigation of the evil. First, it may be asked, whether legislation can do any good. There is great doubt whether good can come of endeavouring to make people virtuous by acts of parliament. There is the old adage, *Leges sine moribus non prosunt.* We dare not go so far as the hope of Mr. Acton, that the day may come when the communication of syphilis to a minor may be made a felony (page 130). Nor is it so clear that all the centralisation of despotic bureaucracy is a match for the spread of licentiousness. If we are to believe all accounts of the doings in Paris, in Naples, in Madrid, and in nearly all the capitals of Europe, we shall have to bewail more over the degeneracy of morals in general, than of London in particular. And if we compare the prevalence of venereal disease in military hospitals of different countries, we find that notwithstanding all police and medical arrangements, our *laisser aller* system, as it is called, is not so prejudicial as might have been expected. Thus of British troops we learn that—

"For seven years and a quarter previous to 1837, the annual number of venereal diseased cavalry was 181 per 1,000, and from 1837 to 1847, 206 per 1,000; while at Brest, the Portsmouth of France, out of 5,947 men, forming in 1852 the marine artillery and infantry of the garrison, 1,635 or 27·50 per cent contracted syphilis. In 1853, out of an effective of 6,294 individuals, 2,144, or 34 *per cent.* were attacked." In Brussels, "of a garrison of less than 3,000 men, the entries into the military venereal hospital were 413 in the year 1856. On the other hand, American military reports are more favourable, being on an average only one venereal case to 35 men." (p. 39.)

Thus, under rigid rule, civil and military, there is no encouragement for drawing tighter the reins accorded in England to individual liberty. We extract from page 79 some examples which our continental neighbours deem requisite for the preservation of decency in disgrace. Every registered woman of the town in Paris is furnished with a sort of "ticket-of-leave," which may be understood as a government recommendation and guarantee of the sanitary excellence of the commodity for disposal, (*breveté par S. M. I.*, would be the proper description). On one side of this ticket is the name, with the woman's age, appearance, residence, &c., with the fortnightly signature of the medical inspector. On the other side of the card are printed the rules:—

"Obligations and Restrictions on Public Women—

"Public women, *en carte,* are called upon to present themselves at the dispensary for examination once at least every fifteen days.

"They are called upon to exhibit this card on every *request of police officers and agents.*

"They are forbidden to practise their calling during daylight, or to walk in the thoroughfares until at least half an hour after the public lamps are lighted, or at any season of the year before seven o'clock or after eleven p.m.

"They must be simply and decently clad, so as not to attract attention by the richness, striking colours, or extravagant fashion of their dress."

Many more regulations follow which we cannot afford space to copy, but simply state that they attempt to provide a certain amount of respectable exterior in the carrying out of an infamous traffic, while at the same time they endeavour to control disease. We doubt on the whole whether either of these results is obtained.

This system once prevailed in England, but we suppose it has become obsolete from its impractical value. For, on referring to *Stow's Survey of London*, 1603, we find a notice of the stews on the bank of the Thames :

" Next on this bank was sometimes the Bordellos or Stewes, a place so called of certain stew-houses privileged there, for the repair of incontinent men to the like women, of the which privilege I have read thus.

"In a parliament holden at Westminster the 8th of Henry II, it was ordained by the commons, and confirmed by the king and lords, that divers constitutions for ever should be kept within that lordship or franchise, according to the old customs that had been there used time out of mind, amongst the which these following were some :—

" 'That no stew-holder or his wife should let or stay any single woman to go and come freely at all times when they listed.

" 'No stew-holder to keep any woman to board, but she to board abroad at her pleasure.

" 'To take no more for the women's chamber in the week than fourteen pence.

" 'Not to keep open his doors upon the holidays.

" 'Not to keep any single woman in his house on the holidays, but the bailiff to see them voided out of the lordship.

" 'No single woman to be kept against her will, that would leave her sin.

" 'No stew-holder to receive any woman of religion, or any man's wife.

" 'No single woman to take money to lie with any man, but she lie with him all night till the morrow.

" 'No man to be drawn or enticed into any stew-house.

" 'The constables, bailiff and others, every week to search every stew-house.

" 'No stew-holder to keep any woman that hath the perilous infirmity of burning, not to sell bread, ale, flesh, fish, wood, coal, or any victuals, &c.'

"These and many more orders were to be observed upon great pain and punishment. I have also seen divers patents of confirmation, namely, one dated 1345, the 19th of Edward III.* Also I find that, in the 4th of Richard II, these stew-houses, belonging to William Walworth, then mayor of London, were farmed by Froes of Flanders, and spoiled by Wat Tyler and other rebels of Kent. Notwithstanding, I find that ordinances for the same place and houses were again confirmed in the reign of Henry VI, to be continued as before. Also Robert Fabian writeth, that in the year 1506, the 21st of Henry VII, the said stew-houses in Southwarke were for a season inhibited and the doors closed up, but it was not

* Li St. Mary Eborum. " English people disdayned to be baudes. Froes of Flaunders were women for that purpose." STOW.

long, saith he, ere the houses there were set open again, so many as were permitted; for, as it was said, whereas before were eighteen houses, from thenceforth were appointed to be used but twelve only. These allowed stew-houses had signs on their fronts towards the Thames, not hanged out, but painted on the walls, as a Boar's Head, The Cross Keys, The Gun, The Castle, The Crane, &c."

In a note by Wm. J. Thoms, Esq., Secretary of the Camden Society, in his edition of Stow, he adds:

"The class of persons referred to in the text became the subject of legislative enactment in this country, apparently at a much earlier period than in France, where their numbers appear to have increased so greatly during the reign of St. Louis as to have called forth an extremely severe ordinance for their regulation, which is quoted by Roquefort in his *Glossaire*, s. v. *Femmes foles de leur corps*. See also Legrand d'Aussy, *Fabliaux ou Contes*, iv, p. 217, *ed.*, 1829."

Seeing then that legislative enactments have never proved of much value in limiting the progress of the disease, it would hardly be possible, in the present tone of the public mind, to revive laws which might be considered entrenching on the liberty of the person. It is to be considered whether our police are such patterns of morality and integrity, as to be entrusted with such a power of annoyance as that of demanding, at all times, a view of the registered card of an unfortunate woman. It is impossible to deny that this publicity attached to individuals by registration of their profession, would be a lifelong and ineradicable disgrace, and would only make those thus circumstanced more reckless and abandoned. As Mr. Acton asserts that most of these women, after three or four years dissipation, re-enter the ranks of the ordinary population, and as their main hope will ever be that their past life may be forgotten, we cannot help thinking that the continental system of registration and surveillance, would not only be rejected by English females, but would be found utterly impracticable by our government. Nor does it seem clearly established why their diseases should claim a special governmental care. It has ever been the admiration of foreigners, that our hospitals are supported by voluntary contributions; and we have yet to learn that, on the continental plan, the patients have either better care, or better skill than what they have in England. It is also suggested by the author, either that the power of domiciliary visitation should be carried out by the district medical officers of health, or that there should be divisional surgeons and inspectors. We cannot think that women would in general submit to this inquisitorial system. However little the public may think of the *femme galante*, she is by no means disposed to think as little of herself. Although she may lack modesty in one particular, she is by no means willing to be treated with less deference and respect than that which her education, her intelligence, her manners, or her good looks will command for her. As for a self-supporting sanitary society, there is but little possibility of any such scheme being carried out. Ladies for whom such a "benefit society" is intended, are habitually improvident, and if not so, would scarcely care to invest money in a sick club, any more than in a savings' bank. We are told that at the Royal Free Hospital more cases of venereal disease are relieved, than at any other hospital in London. And this is not to be wondered at, because, at that hospital, no recommendation from governors is required.

It does seem that the only practical way of doing any good to the unfortunate who may be suffering from this disease, would be to increase the hospital and dispensary accommodation for this especial complaint, and to let it be on the same principle as that of the Royal Free Hospital. Start a hospital or dispensary for all comers, and let there be no historical questions asked of the miserable sufferers; let disease be the simple letter of recommendation, and then, no doubt, the sick will avail themselves of such a charity with heartfelt gratitude. No one likes to publish his own disgrace: then why should we expect that women should prove an exception? Nay, rather, we think, would they suffer disease and death, than let all the world know their degradation. We cannot therefore hope, nor indeed could we wish, that Englishwomen, however low they may have descended in their habits, would yet voluntarily enrol themselves either as professional prostitutes, or as members of a prostitutes' mutual aid and sanitary society.

Whether from the increasing decay of morals, or whether from the publicity now given by the press to all the vices of the age, it is certain that great uneasiness is felt by all thinking people at the progress which vice seems to be making amongst us; and people are naturally anxious, if only for self-preservation, that some check should be placed on its development. Neither laws nor punishments can ever supply the place of sound moral training; and all the centralisation in the world will never reform an iniquitous age. Concubinage, which is but a substitute for polygamy, is generally considered as a token of national decline.

There is an evil, possibly even greater than that which has been called the greatest of our social evils; and that is, the consummate class selfishness which prevails. This feeling recognises only the elect, and abandons the rest to reprobation. *Respectability* is the name under which this principle is personified and worshipped. Her votaries live within a charmed circle, comfortably supplied with everything requisite for their physical and spiritual wants. They have good tables, and soft seats in the place of worship. Well dressed, well gloved, and well scented, they carry prayer-books bound in curious antique; they behave decorously, make all responses, and sing *sotto voce.* Well may they congratulate themselves! The votary of *Disreputability* acknowledges that her goddess has a brazen impudent carriage, but says she is no more than what she professes to be, whilst she accuses Respectability of being little better than a solemn mockery. Open blackguardism is what she prefers, rather than organised and conventional hypocrisy.

We must conclude this notice of Mr. Acton's book, and pay a tribute to his courage in bringing before the British public the dark side of their social condition. It must evidently tend to ameliorate this state of things. What are Church and State about, that they take no heed of the times? Have they adopted the motto, *Après moi le deluge?* We shall see.

---

